# Practical Points in Nursing for Nurses in Private Practice

**Published:** 1899-11

**Authors:** 


### Books and Magazines.

PRACTICAL POINTS IN NURSING FOR NURSES IN PRIVATE PRACTICE. With an appendix containing Rules for Feeding the Sick; Receipts for Invalids Foods and Beverages; Weights and Measures; Dose List; and a full glossary of medical terms on Nursing treatment. By Emily A. M. Stoney, graduate of the Training School for Nurses, Carney Hospital, South Boston. Illustrated with seventy-three engravings in the text, and nine colored and half-tone plates; 456 pages. Price, $1.75 net. Philadelphia: W. B. Saunders, 925 Walnut St.

This volume is written in popular language, and explains the difference between private nursing and hospital nursing. It teaches how best to meet the various emergencies "when distant from medical or surgical aid, or when thrown on her own resources, studiously refraining, however, from advising the nurse to act upon her own responsibility or to assume personal treatment of the patient, except under circumstances of great emergency."

The text of the book is divided into the following:

I. The Nurse; her responsibilities, qualifications, equipment, etc.

II. The sick room; its selection, preparation and management.

III. The patient; duties of the nurse in medical, surgical, obstetrical and gynecological cases.

IV. Nursing in accidents and emergencies.

V. Nursing in special medical cases.

VI. Nursing of the new-born and sick children.

VII. Physiology and descriptive anatomy. B.

